# Addressing Missed Opportunities for Vaccination among Children in Hospitals: Leveraging the P-Process for Health Communication Strategies

**DOI:** 10.3390/vaccines12080884

**Published:** 2024-08-03

**Authors:** Baldeep K. Dhaliwal, Joseph L. Mathew, Patience N. Obiagwu, Rachel Hill, Chizoba B. Wonodi, Tyler Best, Anita Shet

**Affiliations:** 1 Department of International Health, Johns Hopkins Bloomberg School of Public Health, Baltimore, MD 21205, USA; cwonodi1@jhu.edu (C.B.W.); ashet1@jhu.edu (A.S.); 2 International Vaccine Access Center, Johns Hopkins Bloomberg School of Public Health, Baltimore, MD 21205, USA; rachelwilliamshill@gmail.com; 3 Advanced Pediatrics Centre, Post Graduate Institute of Medical Education and Research, Chandigarh 160012, India; dr.joseph.l.mathew@gmail.com; 4 Department of Pediatrics, Aminu Kano Teaching Hospital, Kano 700101, Nigeria; patience.obiagwu@gmail.com; 5 Center for Communication Programs, Johns Hopkins Bloomberg School of Public Health, Baltimore, MD 21205, USA; tylerbest@jhu.edu

**Keywords:** missed opportunities for vaccination, MOV, human-centered design, qualitative research, health communication, P-Process

## Abstract

Addressing missed opportunities for vaccination requires a nuanced and context-specific approach. The five-step P-Process provides a robust framework to develop a clearly defined strategy that addresses social and behavioral drivers, integrates into existing health delivery systems, and facilitates collaboration with local experts. This approach allows teams to design, implement, monitor, and evaluate strategies to address public health issues. However, its specific application in vaccination communication programs remains relatively underexplored and under-documented. Our team designed a multi-pronged communication intervention aimed at enhancing vaccine uptake among hospitalized children in two tertiary hospitals in India and Nigeria. In the Inquiry stage, we conducted in-depth interviews with caregivers of hospitalized children to assess barriers to vaccination in a hospital setting. In the Strategic Development stage, we developed a blueprint for activities, identifying target audiences and communication channels and developing implementation plans. During the Create and Test stage, we brought together a range of stakeholders to co-develop a communication intervention through human-centered design workshops, after which we piloted the materials in both hospitals. We then Mobilized and Monitored progress of the activities to identify potential gaps that our materials did not initially address. Lastly, in the Evaluate and Evolve stage, we conducted in-depth interviews with healthcare workers and caregivers to measure outcomes and assess the impact on caregivers’ decisions to vaccinate their hospitalized children. By following the P-Process for the design, caregivers reported that many of their concerns about vaccines were alleviated, and HCWs reported that they were able to communicate with caregivers more effectively about vaccination. By harnessing the power of the P-Process, researchers can forge a context-specific path towards impactful vaccination communication interventions, one step at a time.

## 1. Background

Vaccination has faced a range of challenges including growing vaccine hesitancy, misinformation, inequitable access, and political motivations and inaction [1]. In addition to these challenges, missed opportunities for vaccination (MOVs) have emerged as a key issue to be addressed to facilitate improved vaccine uptake [2,3]. A missed opportunity for vaccination refers to any contact with the health system by an individual who is eligible for vaccination but does not receive the doses for which they are eligible [2]. Global immunization goals have been stalled due to the COVID-19 pandemic for a range of reasons [4,5,6], and addressing MOVs is a key strategy to resume progress towards these objectives. The prevalence of MOVs varies across countries, with some settings reaching up to 89% [1]. As such, addressing MOVs has become essential to meeting vaccination goals and beginning to undo the pandemic’s damage on immunization progress. 

**Figure 1:**
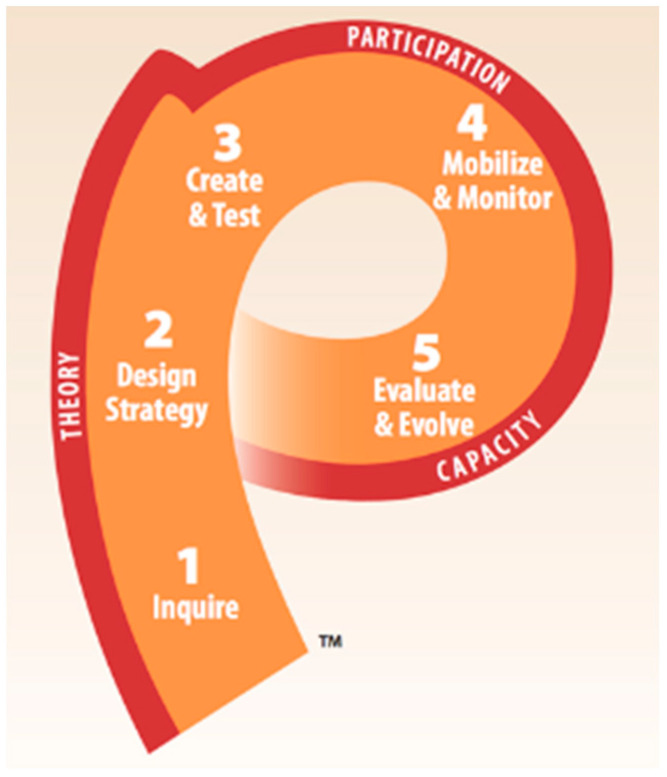
The P-Process. Image Source: Johns Hopkins Bloomberg School of Public Health/Center for Communication Programs.

To make any improvements in public health, it is essential to design effective messaging, as this can have a positive impact on the health of community members [7,8,9]. Similarly, ineffective public health messaging can lead to higher stress and stigma [10,11] and cognitive biases [12,13,14]. The P-Process (Figure 1) is a comprehensive five-step plan to design effective public health messaging [15,16]. This process is designed to guide individuals from the initial idea of ‘changing behavior’, to a well-planned communication campaign that facilitates, monitors, and evaluates behavior change [17]. It is one of the most well-recognized tools, integrating three well-known, cross-cutting concepts into each step: *social and behavior change communication (SBCC) theory*, *stakeholder engagement*, and *continuous capacity strengthening*. Historically, this approach has been instrumental in public health efforts to reduce HIV transmission, promote family planning, reduce infant and maternal mortality, prevent infectious diseases, and protect the environment [16,18]. Based on its extensive use in public health efforts, the P-Process is a highly effective strategy to address MOV. 

In the Inquire Stage (Step 1), the public health problem is assessed through formative and baseline research by analyzing local knowledge, attitudes, and practices and reviewing existing data and policies. Insights from this step inform a situational analysis that outlines the problem, causes, and potential solutions. During the Design Strategy stage (Step 2), stakeholders collaboratively develop a strategic plan, deciding on target audiences, interventions, objectives, communication channels, and implementation plans. In the Create and Test stage (Step 3), stakeholders co-develop and pilot communication materials. The Mobilize and Monitor stage (Step 4) involves deploying communication materials and assessing their effectiveness in real time. Finally, in the Evaluate and Evolve stage (Step 5), the impact of the communication efforts is assessed, and results are shared to guide future initiatives and facilitate capacity building [16].

Our team followed the P-Process to develop a multi-pronged communication intervention aimed at increasing vaccine uptake among children admitted to two tertiary hospitals in Chandigarh, India, and Kano State, Nigeria. By utilizing the P-Process, we developed a compelling communication package of materials to engage healthcare workers (HCWs) and caregivers (parents) of children, empowering them to make more informed vaccination decisions for children admitted to tertiary-care hospitals. This approach allowed us to effectively engage with a wide range of stakeholders, apply SBCC strategies to promote social and individual change, foster an environment that facilitates this change, and, ultimately, enable capacity strengthening in these settings. 

Although the P-Process is well established in several public health domains (i.e., HIV, family planning) [16,18], its application to vaccination communication interventions has been comparatively under-documented in research settings. In this paper, we describe how the P-Process facilitated the design of a communication intervention, implemented as part of a larger study on MOVs [2]. 

We aim to provide a detailed description of the structured process used to identify, develop, pilot, and evaluate communication solutions to address MOVs. While it is common to read about the implementation or outcomes of communication interventions, it is less common to read the systematic processes used to create these interventions. The P-Process is a structured approach for developing broad public health communication interventions, which has rarely been documented in the literature for its role in designing facility-based communication interventions. We aim to fill this gap in communication research by providing both researchers and practitioners with guidance for using the P-Process within the context of a research project.

## 2. Methods

### 2.1. Setting

This research was conducted by the International Vaccine Access Center (IVAC) at the Johns Hopkins Bloomberg School of Public Health (JHSPH) in two hospitals in Chandigarh, India, and Kano State, Nigeria. The IVAC is widely recognized as a center of international excellence and innovation in epidemiology, health policy, health economics, public health, advocacy, and research. The research team at the IVAC collaborated with the Post Graduate Institute of Medical Education and Research (PGIMER), a premier medical and research institution in Chandigarh, India. PGIMER is a leading tertiary-care, 2000+ bed hospital, drawing in patients from states across Northern India. Additionally, the IVAC team collaborated with the Aminu Kano Teaching Hospital (AKTH) in Kano, Nigeria, a tertiary-care, 500-bed hospital in the most populous state in Nigeria. 

### 2.2. Ethics

We obtained ethical approval from the JHSPH Institutional Review Board (#11429), the Institutional Ethics Committee of PGIMER, Chandigarh (IEC-11/2019-1431), and the National Health Research Ethics Committee of Nigeria (NHREC/01/01/2007). We obtained written consent from all participants prior to data collection. As this study was conducted during the height of the COVID-19 pandemic, we designed a safety plan to protect both study staff and study participants from COVID-19. We provided and required personal protective equipment, provided face coverings to participants, limited the duration of interactions, ensured data were collected outdoors as much as possible, and we implemented a cleaning protocol where appropriate. 

### 2.3. Study Design Overview

We leveraged the P-Process as part of a larger study focused on MOVs among hospitalized children. The primary objective of this larger study was to quantify the proportion of under-vaccinated children who do not receive any catch-up vaccines at the time of hospital discharge [19]. The overall study aimed to develop, pilot, and propose broader potential solutions to address the problem of missed opportunities among hospitalized children. The steps of the P-Process were carried out in both hospitals in India and Nigeria.

### 2.4. Nested Study Design and Description

**Figure 2:**
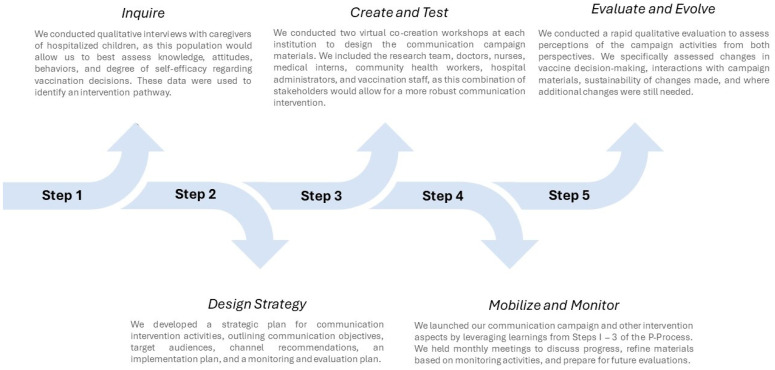
Application of the P-Process for a communication intervention to address MOVs.

To create a communication intervention and other activities to address MOVs, we involved stakeholders using the five-step P-Process to design, implement, monitor, and evaluate our activities (Figure 2). Stakeholders included the research team, a range of diverse healthcare workers at the hospital sites, administrative and immunization staff, and community health workers. 

#### 2.4.1. Step 1: Inquire

The research team began by identifying participants for our baseline research. We conducted in-depth interviews (IDIs) with caregivers of hospitalized children to assess the barriers and facilitators to accessing routine vaccination services in tertiary-care hospitals. This population was selected as they allowed us to best assess the knowledge, attitudes, behaviors, and degree of self-efficacy regarding vaccination decisions for hospitalized children. The interview guide included open-ended questions on caregiver perceptions of vaccines, vaccine decision-making processes, available and impactful communication channels, and the impacts of COVID-19 on vaccination experiences. Local data collectors conducted interviews between August and September 2020 in Hindi or Punjabi at PGIMER and in Hausa at AKTH. Trained research personnel interviewed eleven caregivers at PGIMER and thirteen caregivers at AKTH prior to reaching saturation. Our team assessed that we reached saturation when we had sufficient data to assess patterns and themes across the transcripts. Interviews were conducted in person at the hospitals, and they lasted between 30 and 60 min. Interviews were recorded, transcribed by an external transcription company, and reviewed by the original interviewers to confirm accuracy. All personal data were removed to secure participant anonymity. 

#### 2.4.2. Step 2: Design Strategy

After completing baseline data collection, our research team developed a strategic blueprint for communication and intervention activities. This strategy outlined communication objectives, target audiences, communication channels, and a monitoring and evaluation plan. While the data collected in Step 1 informed the strategy, we identified the need for two virtual human-centered design (HCD) workshops to further refine the strategic plan [20,21]. HCD was selected as data have demonstrated that HCD-derived strategies may be more efficacious and impactful in real-world settings, particularly compared to strategies developed through other approaches [22,23,24]. Further, we chose to use virtual platforms to conduct HCD, as using web-based platforms to conduct research has been found to be an effective strategy to combat systemic barriers associated with in-person activities—particularly in hierarchical settings—such as power, economic, social, and gender dynamics [25].

#### 2.4.3. Step 3: Create and Test

Given the iterative nature of HCD, and its focus on human engagement and stakeholder-responsive design [20], we determined that HCD was well suited for this stage of the P-Process. With an understanding of the barriers and facilitators, and a strategic plan in place, we conducted online HCD workshops at each institution in November 2020 to create our materials [21]. These two-hour workshops, conducted on virtual conferencing platforms, included the research team, doctors, nurses, medical interns, community health workers, hospital administrators, and immunization clinic staff. These diverse participants helped design a more robust communication intervention. 

**Figure 3:**
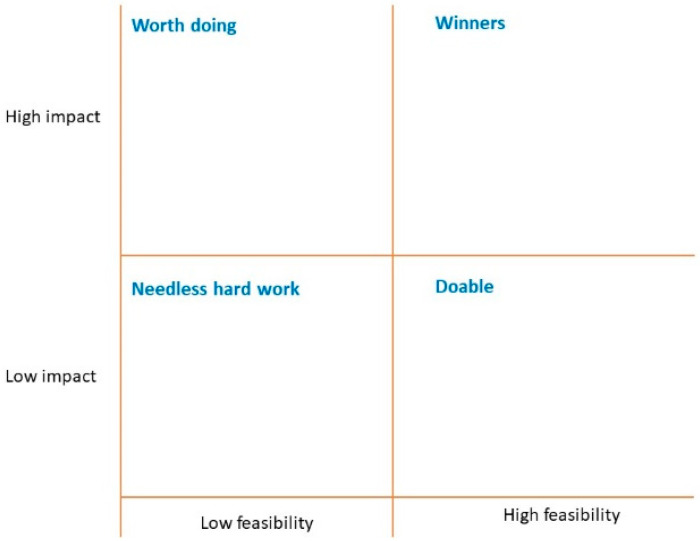
Priority matrix.

The research team presented preliminary findings from the Inquire step to participants attending the HCD workshops. In breakout group discussions, participants collaborated to develop a communication intervention and other initiatives. These discussions allowed us to co-create strategies to overcome identified barriers, identify content materials, determine optimal material display, assign responsibility for additional tasks, and ensure the sustainability of changes. After the breakout sessions, participants used a ‘priority matrix’ to rank the impact and feasibility of each proposed communication aspect (Figure 3). The workshops resulted in two distinct prototypes to motivate routine vaccine uptake among hospitalized children. These prototypes varied between the settings, as they were tailored to the unique needs of the setting, based on the unique barriers identified in the *Inquire* step as well as the HCD workshops. Involving stakeholders through these workshops was critical to the success of the design of our intervention initiatives. For example, while we had initially discussed the possibility of using posters at both AKTH and PGIMER, stakeholders at PGIMER informed our team that posters would be less effective than other strategies. 

#### 2.4.4. Step 4: Mobilize and Monitor 

After completing the HCD workshops and finalizing our materials, we launched the communication and intervention aspects between December 2020 and January 2021. The communication activities targeted misconceptions and barriers that were identified in the Inquire stage by leveraging the identified communication channels. The research teams held individual and collaborative monthly meetings from initiation through May 2021 to discuss progress, refine materials based on monitoring activities, and prepare for future evaluations. 

#### 2.4.5. Step 5: Evaluate and Evolve

At the end of the implementation period, we interviewed 15 caregivers at each hospital (30 qualitative interviews with caregivers in total), along with eight HCWs at each facility. After conducting these forty-six interviews, our team assessed that we had reached saturation, and we had sufficient data to identify key post-campaign themes. Interviews were conducted in a private area of the hospital between July and August 2021, and they lasted between 30 and 60 min. Caregiver interview domains included the following: changes in vaccination decision-making, changes in vaccine knowledge, interactions with materials, and additional changes needed. The HCW interview guide included open-ended questions on changes in vaccine knowledge, changes in ability to communicate about vaccination, sustainability of materials, and additional changes needed. Interviews were transcribed by an external transcription company, checked for accuracy by the interviewers, and all personal data were removed to protect participant privacy. Subsequently, we conducted rapid thematic analysis on the interview transcripts to assess perceptions on the communication materials, as well as other intervention aspects. We inductively coded our transcripts, allowing the data to guide our analysis process as opposed to starting with an initial framework. After identifying initial codes, we grouped these codes into broader themes to document our learnings.

## 3. Results 

Results from each stage of the P-Process iteratively allowed our team to design, implement, and evaluate our communication intervention to improve vaccine uptake among hospitalized children in two tertiary-care hospitals. 

### 3.1. Step 1: Inquire

We conducted a preliminary analysis of qualitative data by analyzing interview transcripts from PGIMER and AKTH separately. We used interview transcripts to rapidly transform our research data into meaningful and actionable insights to design our activities by (1) organizing insights into meaningful categories; (2) organizing categories into qualitative themes; and (3) preparing these for presentation in future workshops to design possible solutions. Key themes are documented in Table 1. 

### 3.2. Step 2: Design Strategy

After initial baseline data collection and analysis, our team collaboratively developed a strategic plan for communication activities based on learnings in the Inquire stage (Step 1). This strategic plan is detailed in Table 2.

### 3.3. Step 3: Create and Test 

After co-creating the communication activities, we designed and implemented these materials in PGIMER India, and AKTH, Nigeria. These strategies utilized communication materials for caregivers, communication and educational training for HCWs, and strategies to improve communication between HCWs and caregivers. Specifically, these communication activities focused on (1) disseminating information to increase awareness for caregivers, and educating HCWs on how to communicate new knowledge to caregivers; (2) improving communication of vaccination status from HCWs to caregivers; (3) enhancing communication between immunization workers, physicians, and caregivers to improve vaccination delivery to under-immunized children; and (4) providing advice to caregivers upon discharge through educational and informational pamphlets. Specific and tailored materials are documented in Table 3.

### 3.4. Step 4: Mobilize and Monitor 

After initiating our activities, we conducted ongoing monitoring to evaluate progress. This was reviewed during our teams’ monthly meetings to determine if adjustments were necessary. For example, our team determined that after the educational quizzes and emails were shared with the HCWs at PGIMER it would be important to share a physical FAQ document with them. This was based on the perceived needs of the HCWs. During our monthly monitoring meeting, we determined that developing a document that compiled all key points from the emails and quizzes would allow HCWs to more easily access the information. Our activities are documented in Table 4 and Table 5.

### 3.5. Step 5: Evaluate and Evolve 

The analysis of the qualitative interviews identified that caregivers were discussing their child’s vaccination status more frequently while hospitalized, and that their vaccination concerns were more regularly addressed. Our data suggested that HCWs were more diligent in checking a child’s immunization card and were more willing to vaccinate a child while they were hospitalized. Across both populations, we found that materials were beneficial and provided additional vaccine knowledge. HCWs noted that external aids and specific communication strategies—such as stickers in PGIMER and posters at AKTH—served as consistent reminders to ensure that admitted children were up to date on all vaccines. These results served to qualitatively indicate a post-intervention increase in vaccine uptake among the caregivers and HCWs. 

Our analysis also uncovered gaps; despite improved communication between HCWs and caregivers, some vaccine hesitancy persisted among caregivers, and some children discharged from the hospital remained under-vaccinated.

## 4. Discussion

Traditionally, MOVs research in tertiary-care settings has focused on documenting the number of children who contact the health system without receiving catch-up vaccines [26] or documenting the reasons that these children have been missed [27]. There is a dearth of data on strategies to address MOVs [28]; the few available studies are from high-income countries, which may not be generalizable to other settings [29,30,31,32].

Communication research for immunization has traditionally focused on exploring interactions with social media [33,34,35], assessing communication strategies during healthcare visits [36,37,38], and designing and evaluating communication technology and tools for healthcare workers [39,40]. As there are limited data on MOV interventions—as well as the design of communication materials—it is essential to leverage and document approaches that support the design of context-specific strategies, such as HCD and the P-Process.

Designing and Implementing Contextually Relevant Strategies using a Single Methodology: While communication professionals have traditionally used the P-Process to design, implement, and evaluate public health campaigns, researchers too can leverage this comprehensive approach across a range of public health settings. The P-Process methodology allows for strategic, evidence-based, socio-behavioral change initiatives, assisting with driving positive outcomes for communities in different contexts. Significantly, this approach facilitates using a single strategy to design multiple communication campaigns tailored to the unique needs of various settings, as we carried out in both India and Nigeria.

Using Research Insights as a Foundation for Program Development: The insights from conducting formative research serve as the foundation for designing targeted communication materials aimed at addressing the identified issues. By leading rapid, yet rigorous, formative research to develop baseline knowledge before designing and initiating communication campaigns, researchers can quickly turn research insights into effective program ideas before advancing further in the design of a communication campaign or intervention.

Translating Research Findings into Actionable Program Ideas: After completing formative research, researchers can collaborate with local experts to develop specific communication materials and strategies. Academic learnings can be translated into actionable and practical program ideas through strategies like human-centered design and co-creation workshops. In carried this out, researchers would be able to more effectively align program activities with the needs of the target audiences to maximize communication effectiveness.

Maximizing Impact through Evidence-Based Communication: Lastly, implementing activities that are evidence-based, and made in conjunction with the community, have the potential to broadly improve health behavior [41,42]. As vaccination decisions are complex—influenced by family beliefs, access issues, community norms, and political influences—engaging deeply with the target populations to identify barriers and facilitators, actively monitoring progress, and making iterative changes can maximize the impact of communication efforts [43].

This project was limited by several factors. We conducted this project across two hospital sites—each with unique cultural and contextual settings—which may limit the generalizability of our findings. We acknowledge the lack of a control group in assessing our overall outcomes, as we used a quasi-experimental pre–post design. There is a possibility of volunteer bias, particularly among caregivers and workshop attendees, as participants with extreme positive or negative feelings about vaccination might have been more willing to participate. Another limitation is the potential for confirmation bias during the HCD workshops, as we shared preliminary data and we identified possible areas for communication activities. It is possible that attendees did not feel comfortable disagreeing with our preliminary findings or did not feel comfortable sharing new insights. However, we attempted to mitigate this by conducting HCD workshops online, aiming to lessen these power dynamics. Additionally, we conducted formative research in mid-2020, and we conducted evaluative IDIs in mid-2021, both during the height of the COVID-19 pandemic. This could have created an uncomfortable dynamic for caregivers and HCWs, as interviewers were distancing and wearing personal protective equipment, preventing participants from fully engaging in research activities. Further, we conducted our HCD workshops in November 2020 by using a virtual platform to minimize the spread of COVID-19. This may have prevented stakeholders from speaking freely during the workshops, as there may have been connectivity or network issues. Lastly, the COVID-19 pandemic likely influenced general vaccination discussions, as participants may have been particularly eager to access vaccines. The heightened frequency of vaccine-related discussions during this period could have positively influenced our findings.

## 5. Conclusions

Applying the P-Process is an effective approach for designing communication strategies to address a range of public health issues. Its iterative nature allows researchers to develop context-specific strategies tailored to the unique needs of each setting, while acknowledging the constantly evolving nature of individual behavior and decision-making. Leveraging this approach for MOVs in tertiary-care settings was particularly beneficial, as it facilitated an iterative approach to allow for adjustments in response to rapidly shifting beliefs, changing norms, and emerging sources of dis/misinformation—all of which are common in vaccination. Using the P-Process to design iterative communication and intervention activities can enhance the success of future public health initiatives, transforming the landscape of health communication, and, ideally, paving the way to a healthier future for all.

## Figures and Tables

**Table 1:** Qualitative baseline findings from India and Nigeria.

Key Finding	Detail	Quote
Caregiver Knowledge Barriers	Caregivers of hospitalized children believed that their child could only be vaccinated if they were in good health. This misinformation was further reiterated by healthcare workers.	*“Ma’am, there are ASHA workers who call us. They ask us first about a child’s health like whether he is having fever, cold, or diarrhea. Then we tell them he is like this—then they decide about the vaccination. They say when your child will be completely normal, then he will get vaccinated.”—Caregiver, India* *“Based on the explanation of doctors, it is not advisable for a child to be immunized when he is sick. I have to wait until the child health is improved and he is back to normal.”—Caregiver, Nigeria*
Caregiver Access Barriers	Caregivers of hospitalized children were unsure about their ability to get their children vaccinated due to the pandemic.	*“Local hospitals are COVID-only hospitals now, so [we are] not sure on where to go.”—Caregiver, India* *“You might not be attended to—except when you go to [the] emergency [department]. And once it is 5 pm, they are closed for the day. Because of COVID-19, they have reduced the number of patients to be attended [to].”—Caregiver, Nigeria*
Sources of Information	Caregivers shared that, in addition to medical workers, there were other individuals who played a major role in vaccination decisions.	*“They [Anganwadi workers] give us information about those things. They say that there will be a short-term problem but in the long term, your child will be very healthy. Then we think that we should get our child vaccinated, because it’s the question of the child’s life.”—Caregiver, India* *“I have conversations with my neighbor a lot. Honestly, she is even the one that suggested a hospital close to us. She said they administer vaccines there. That is where all her children got immunized. I told her I will go there. She even offered to accompany me if I was reluctant.”—Caregiver, Nigeria*

**Table 2:** Strategic plan for communication activities.

Strategic Activity	Details
Target Audiences	We determined that caregivers and HCWs should be the target audience for the communication aspects. While certain aspects of our approach would target caregivers directly, other aspects were geared towards HCWs to facilitate improved communication.
Communication Objectives	Provide educational and communication training to HCWs about contraindications to vaccines. Disseminate accurate vaccine information to caregivers through both HCWs and materials. Improve communication between caregivers and HCWs to effectively communicate when children are eligible for vaccination. Target communication activities to caregivers and other common sources of information. Improve communication to caregivers about accessing vaccination services after hospital discharge, despite the COVID-19 pandemic.
Communication Channels	We determined that a multi-pronged approach would be most effective at reaching the widest audience. This included placing posters in frequently visited locations for caregivers and HCWs, emailing critical information to HCWs, conducting in-person training sessions for HCWs on vaccine knowledge and communication, and disseminating pamphlets to caregivers upon discharge.
Implementation Plan	Our team decided that two co-creation workshops would best facilitate the design of our activities. After initiating the communication activities, we planned to implement changes for at least four months in each hospital; we determined that monthly meetings to review progress and make iterative changes would be most beneficial. Finally, we planned to assess the impact of the intervention through qualitative IDIs with caregivers and HCWs.
Monitoring and Evaluation Plan	Our monthly monitoring plan included documenting types of communication training activities, the number of session attendees or recipients of information, and the number of children who were provided with vaccination advice before discharge. We planned a final evaluation to assess both HCWs’ and caregivers’ perception of communication activities.

**Table 3:** Communication materials for PGIMER and AKTH.

Communication Materials for PGIMER, India	Communication Materials for AKTH, Nigeria
Educational emails to healthcare providers on contraindications for vaccination. Quizzes for HCWs on contraindications for vaccination and how to communicate vaccination information. Color-coded stickers on hospital admission files to indicate to HCWs whether a child was fully, partially, or unvaccinated, to ensure that they were able to advocate for vaccination services to the caregiver appropriately. Discharge pamphlets for caregivers communicating the child’s current vaccination status and where additional vaccines are accessible.	Teaching session for doctors and medical trainees to provide training on identifying under-vaccinated children and communication strategies. Posters in staff wards providing education and guidance on communicating with caregivers about vaccination services. Posters in Hausa in waiting areas for caregivers providing education on vaccination services and informing caregivers that vaccines during hospitalization are safe. Discharge pamphlets for caregivers communicating vaccine information and where additional vaccines are accessible.

**Table 4:** Monthly monitoring (India).

Monthly Monitoring—PGIMER, India				
Educational Emails—HCWs	February 2021	March 2021	April 2021	May 2021
	215	N/A	N/A	N/A
Educational Quizzes—HCWs	42	N/A	N/A	N/A
Color-Coded Stickers to Indicate Vaccination Status to HCWs (per month)	52	97	51	83
Discharge Pamphlets for Caregivers (per month)	117	32	135	112

N/A: non-applicable.

**Table 5:** Monthly monitoring (Nigeria).

Monthly Monitoring—AKTH, Nigeria					
	December 2020	January 2021	February 2021	March 2021	May 2021
Educational Sessions—HCWs	50 attendees	N/A	47 attendees	N/A	N/A
Posters—Caregivers	6 posters	N/A	N/A	N/A	N/A
Posters—HCWs	6 posters	N/A	N/A	N/A	N/A
Discharge Pamphlets for Caregivers (per month)	9	14	13	8	3

N/A: non-applicable.

## Data Availability

The original contributions presented in the study are included in the article, further inquiries can be directed to the corresponding author/s.
